# Analysis of Gaseous By-Products of CF_3_I and CF_3_I-CO_2_ after High Voltage Arcing Using a GCMS

**DOI:** 10.3390/molecules24081599

**Published:** 2019-04-23

**Authors:** Phillip Widger, Abderrahmane (Manu) Haddad

**Affiliations:** Advanced High Voltage Engineering Research Centre, School of Engineering, Cardiff University, The Parade, Cardiff CF24 3AA, UK; haddad@cardiff.ac.uk

**Keywords:** Trifluoroiodomethane (CF_3_I), Sulphur Hexafluoride (SF_6_), High Voltage Gas Insulation, Gas Insulated Lines (GIL), Gas Insulated Switchgear (GIS)

## Abstract

Increasing demand for an alternative insulation medium to sulphur hexafluoride (SF_6_) has led to the investigation of new environmentally friendly insulation gases which could be used in high voltage equipment on the electrical power network. One such alternative, which is currently being explored by researchers, is Trifluoroiodomethane (CF_3_I) which could potentially be used in a gas mixture with carbon dioxide (CO_2_) as an insulation medium. In this paper an analysis of gaseous by-products detected as a result of high voltage breakdown through pure CF_3_I and a CF_3_I-CO_2_ gas mixture across a sphere-sphere electrode arrangement is given. Gas chromatography and mass spectrometry (GCMS) is used to identify the gaseous by-products produced as a result of high voltage arcing which causes the gas between the electrodes to dissociate. Analysing these gas by-products helps to identify the long-term behaviour of the gas mixture in high voltage equipment.

## 1. Introduction

In the power industry, sulphur hexafluoride (SF_6_) is increasingly being used as a high voltage insulation medium in gas insulated lines (GIL) for power transmission purposes and in gas insulated switchgear (GIS) as an insulating and arc interrupting medium [1]. However, the use of SF_6_ is receiving a lot of scrutiny from the international community as a gas of extremely high global warming potential (GWP) which is estimated to be 23,500 times that of CO_2_ [2] when released into the atmosphere. SF_6_ is an extremely damaging global warming gas with a long atmospheric lifetime of 3200 years [3]. 

At present the international scientific community is searching for an alternative insulation medium which has all the necessary qualities required for use in the power industry, similar to SF_6_, without the damaging environmental effects. At present the candidates being proposed include: Fluoronitrile [4], Fluoroketones [5], Hydrofluoroolefins HFO’s [6] and Trifluoroiodomethane (CF_3_I) [7,8,9] all of which exhibit a lower global warming potential than SF_6_ and a reduced atmospheric lifetime. However, there are still concerns regarding their toxicity and much research is still needed to evaluate their characteristics in full [10,11,12]. In the power industry, at high voltages of 11 kV and above, SF_6_ gas insulated equipment is used in GIS and GIL. Long term partial arcing or full HV breakdowns to ground, in this gas insulated equipment, release large amounts of energy through the insulation gas, which leads to the gas dissociating. This irrevocable gas dissociation can affect the overall insulation strength and operational withstand level of the HV equipment. As such, these events are of real concern to the long-term operation of future alternatively insulated gas equipment. It is important to understand the effect this may have on the gas being used to insulate equipment and whether the gaseous by-products will affect the ability of the equipment to operate safely or pose future health or environmental problems. In this paper the by-products produced from the use of a CF_3_I-CO_2_ gas mixtures are analysed when a high voltage breakdown event is intentionally used to age the insulation gas. In order to fully analyse the behaviour of a CF_3_I-CO_2_ gas mixture, the results have been compared to that of the same practical test undertaken with pure CF_3_I and pure CO_2_ in order to understand how a gas mixture develops by-products compared to its pure counterparts. 

## 2. Results

### 2.1. Results of Pure CO_2_ Gas By-Product Analysis

**Figure 1:**
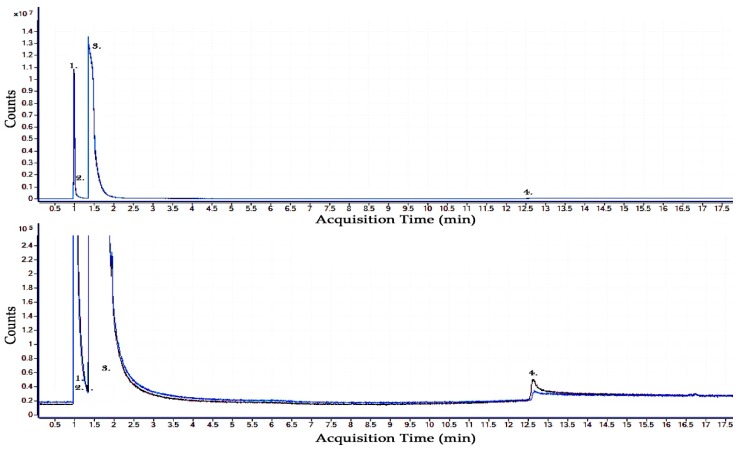
GCMS Analysis of the gaseous by-products of CO_2_, purple—gas sample taken before breakdown, blue—gas sample taken following 100 high voltage AC breakdowns. Detected gaseous molecules: 1. CO—Carbon Monoxide, 2. O_2_—Oxygen, 3. CO_2_—Carbon Dioxide, 4. H_2_O—Water.

The typical breakdown voltage and current level used during the practical testing of the pure CO_2_ sphere-sphere gas gap was approximately 33 kV (peak) and 30 A. An initial gas sample (before HV arcing) is shown in Figure 1. Following 100 breakdowns across the sphere-sphere electrode gas gap, the resulting gas analysis can be shown in Figure 1 along with the original gas sample shown in the same figure for comparison. It can be shown from this figure that after the 100 arcing events, from the energy level available from this transformer, that the CO_2_ molecule is either remaining in the same form or producing solid carbon deposits with a gaseous CO/O_2_ by-product but it is difficult to show this as CO/O_2_ exists in the original samples. No other by-products are detected in a quantity that would allow for the GCMS to conclusively prove their existence. It can be noted that CO_2_, as a relatively simple molecule, remains largely unchanged by the breakdown phenomena it is subjected and therefore can be considered a good choice as a buffer gas for use in insulation gas mixtures. 

### 2.2. Results of Pure CF_3_I Gas By-Product Analysis

Next a pure gas sample of CF_3_I is analysed using the GCMS to compare gas samples both before and after 100 breakdowns across a sphere-sphere gas gap. It can be noted that even after 15 different flushing cycles of the test arrangement and gas sampling system with Helium that residual CO_2_ is still detected by the GCMS due to its level of sensitivity, which is necessary because of the amount of by-products produced. It can also be noted that CHF_3_, C_2_HF_5_ and H_2_O are also present in the pure gas before any breakdown takes place, indicating that these slight impurities exist in the original gas sample or that the gas is reacting with one or more of the storage/test vessel materials. H_2_O likely exists in small quantities due to the fact that the level of vacuum used, which is the normal level for gas insulated power equipment, is insufficient to remove all of the H_2_O from the pressure vessel materials. 

**Figure 2:**
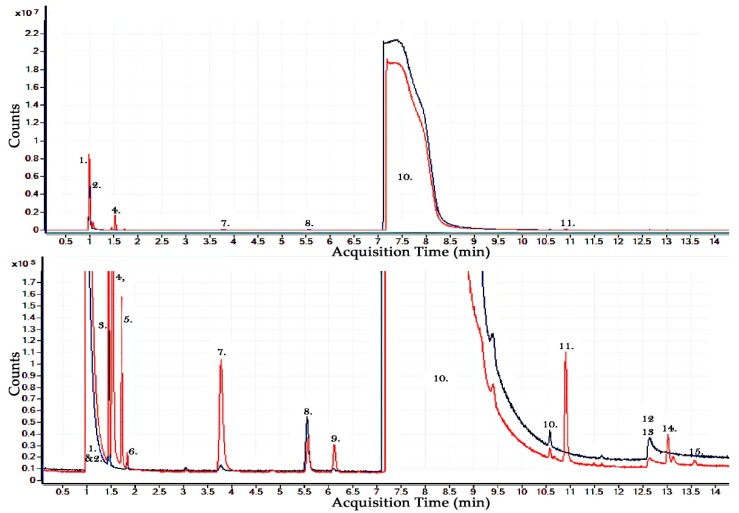
GCMS Analysis of the gaseous by-products of pure CF_3_I, blue—gas sample before breakdown, red—gas sample following 100 high voltage AC breakdowns. Detected before breakdown: 1. CO—Carbon Monoxide, 3. CO_2_—Carbon Dioxide, 6. CHF_3_—Fluoroform, 8. C_2_HF_5_—Ethane, pentafluoro, 10. CF_3_I—Methane, trifluoroiodo-, 12. I_2_—Iodine, 13. H_2_O—Water. Detected after breakdown only: 2. CF_4_—Tetrafluoromethane, 4. C_2_F_6_—Ethane, hexafluoro-, 5. C_2_F_4_—Ethene, tetrafluoro-, 7. C_3_F_8_—Perfluoropropane, 9. C_3_F_6_—Propene, hexafluoro- or C_4_F_8_—Cyclobutane, octafluoro-, 11. C_2_F_5_I—Pentfluoroethyliodide, 14. C_3_F_7_I—Perfluoropropyl iodide, 15. C_3_F_7_IO—Tetrafluoro-1 trifluoromethoxy 1-iodoethane.

Following 100 breakdown events it can be shown in Figure 2 that the original gas impurities/by-products remain along with newly created gaseous by-products, detected only after the 100 breakdown events. These detected by-products of pure CF_3_I include: CF_4_, C_2_F_6_, C_2_F_4_, C_3_F_8_, C_3_F_6_ or C_4_F_8_, C_2_F_5_I, C_3_F_7_I and C_3_F_7_IO. It is likely that C_3_F_7_IO is a by-product caused by the interaction of CF_3_I and the very small quantities of CO_2_ that remain or the H_2_O content which is also present before the breakdown sample is taken. It can be noted that because the molecular weight of iodine is much heavier and larger than the other elements that make up the molecule CF_3_I, that all large molecular by-products that contain iodine have a longer retention time from this column than CF_3_I and the by-products that do not contain iodine have a shorter retention time along this column and are detected near the front end of each run. It is also possible that there are by-products which have the same retention time in this column as CF_3_I and therefore are not able to be distinguished from CF_3_I. It can be shown in Figure 2 that the large amount of CF_3_I in the gas sample takes approx. 2 min to pass the detector. 

It is also possible to show that most of these by-products detected after 100 breakdowns are similar to the by-products detected in pure CF_3_I when subjected to PD events as described by M. kamarol, Y. Nakayama, T. Hara, S. Ohtsuka and M. Hikita in reference [13]. This means that the breakdown event method used in this paper and the partial discharge method used in reference [13] are comparable methods of determining both the long term and short-term arcing by-products of CF_3_I.

### 2.3. Results of 30:70% CF_3_I-CO_2_ Gas By-Product Analysis

Following the tests carried out with pure CF_3_I, a pressure-pressure gas mixture ratio of 30% CF_3_I and 70% CO_2_ was examined to show the by-products produced by this gas mixture using the same sphere-sphere test electrode arrangement. It can be shown that, before any breakdown event takes place, the 70% CO_2_ is detected in the gas sample as well as a CO component. Before breakdown, the 30% CF_3_I component of the gas mixture is detected along with a long 2 min retention time, as well as components such as CHF_3_ and C_2_HF_5_. A small amount of H_2_O is also detected before any breakdown event is undertaken, presumable retained during vacuum from the containing vessel wall materials. 

**Figure 3:**
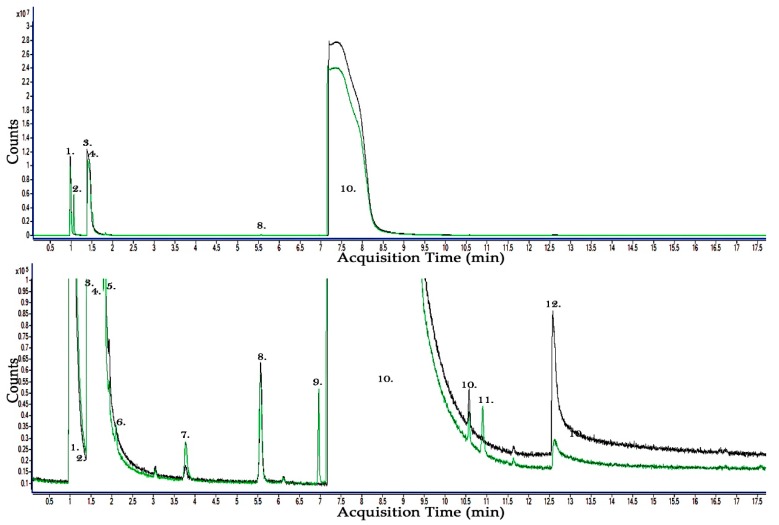
GCMS Analysis of the gaseous by-products of a 30%:70% CF_3_I-CO_2_ gas mixture, black line—gas sample before breakdown, green line—gas sample following 100 high voltage AC breakdowns. Detected before breakdown: 1. CO—Carbon Monoxide, 3. CO_2_—Carbon Dioxide, 5. CHF_3_—Fluoroform, 8. C_2_HF_5_—Ethane, pentafluoro-, 10. CF_3_I—Methane, trifluoroiodo-, 12. H_2_O—Water. Detected after breakdown only: 2. C_2_F_6_O_3_—Trioxide, bis(trifluoromethyl) or CF_4_—Tetrafluoromethane, 4. C_2_F_6_—Ethane, hexafluoro-, 6. C_2_F_6_O_3_—Trioxide, bis(trifluoromethyl), 7. C_3_F_8_—Perfluoropropane, 9. CF_4_—Tetrafluoromethane or C_2_F_6_O_3_—Trioxide, bis(trifluoromethyl) or C_2_H_3_F_3_—Ethane, 1,1,1-trifluoro-, 11. C_2_F_5_I—Pentafluoroethyliodide or C_2_F_9_I—Tetrafluoro(pentafluoroethyl)iodine.

After 100 breakdown events have been carried out across the sphere-sphere electrode gas gap containing 30:70% CF_3_I-CO_2_, a gas sample was taken and the results were analysed using the GCMS as shown in Figure 3. The results show that C_2_F_6_O_3_—Trioxide, bis(trifluoromethyl), C_2_F_6_—Ethane, hexafluoro-, C_3_F_8_—Perfluoropropane and CF_4_—Tetrafluoromethane were detected as by-products from the 30:70% CF_3_I-CO_2_ gas mixture. It is also likely that C_2_H_3_F_3_—Ethane, 1,1,1-trifluoro- and C_2_F_5_I—Pentafluoroethyliodide or C_2_F_9_I—Tetrafluoro(pentafluoroethyl)iodine could also be by-products but these are more difficult to confirm. It should also be noted that C_2_HF_5_—Ethane, pentafluoro- and CHF_3_—Fluoroform were also detected before and after breakdown of this CF_3_I-CO_2_ gas mixture was carried out so they could be potential by-products, however, it is difficult to confirm they are produced as part of the arcing breakdown event and are not merely a by-product of gas interaction with containment materials. More analysis of this result, when compared to pure CF_3_I, is given in the next section of this paper. 

## 3. Discussion

### 3.1. Discussion of Pure CF_3_I and 30:70% CF_3_I-CO_2_ Gas By-Product Comparison

**Figure 4:**
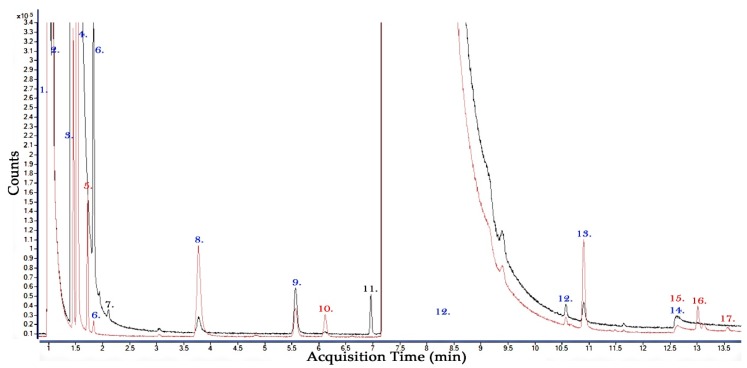
GCMS analysis of the gaseous by-products of a pure CF_3_I and a 30%:70% CF_3_I-CO_2_ gas mixture, red line—pure CF_3_I following 100 high voltage AC breakdowns, black line—30%:70% CF_3_I-CO_2_ gas sample following 100 high voltage AC breakdowns. (Blue numbers—detected molecule in both CF_3_I and CF_3_I-CO_2_ breakdown tests, Red numbers—detected molecules in pure CF_3_I breakdown test only, Black numbered molecules—detected in CF_3_I-CO_2_ breakdown test only) (Analysis of molecule numbers shown in Table 1).

In order to determine which by-products are produced after 100 breakdown events, it is important to examine the pure gases separately and compare these results to the gas mixture result. To identify which by-products are a result of the interaction between the two molecules in the gas mixture, it is useful to compare the analysis of all of these gas samples together i.e. both pure and mixtures. Figure 4 shows the GCMS analysis from both the pure CF_3_I and the 30%:70% CF_3_I-CO_2_ gas mixture results. As pure CO_2_ does not show any pertinent distinguishable results it was not necessary to include it in this discussion. The molecules and by-products detected during the GCMS analysis carried out in Figure 4 are shown in Table 1.

Using the analysis conducted with both pure CF_3_I and CF_3_I-CO_2_ it is possible to identify the by-products that are produced by the CF_3_I component and the alternative/extra by-products that are produced when it is used in a gas mixture with CO_2_. 

It can be shown that prior to the breakdown tests being carried out, in both the pure CF_3_I and CF_3_I-CO_2_ gas samples, that both CHF_3_ and C_2_HF_3_ are detected. This means that these are either impurities in the original gas sample or are a result of interaction with storage/test vessel materials. It is also possible that CHF_3_ and C_2_HF_3_ could be produced as a result of the breakdown arcing tests carried out, however, it is not possible to distinguish these since they are present in the pre-breakdown gas analysis sample. 

From both the pure CF_3_I and CF_3_I-CO_2_ breakdown tests it can be shown that CF_4_, C_2_F_6_, C_3_F_8_ and C_2_F_5_I are produced as gaseous by-products. As these by-products are produced in both tests, this means it is most likely that these by-products are a result of the CF_3_I molecules in the gas mixture only and are not due to any interaction with the CO_2_ in the gas mixture during the arcing event. It is also possible to surmise from this that because the CF_3_I is only 30% of the partial pressure mixture with CO_2_ that the amount of these gaseous by-products will reduce as the amount of CF_3_I gas molecules involved in the arcing event is reduced, however, more research is needed to conclusively prove this. 

In the pure CF_3_I breakdown tests, the gas analysis showed additional by-products of C_2_F_4_, C_3_F_6_, C_3_F_7_I, C_3_F_7_IO and potentially C_4_F_8_ which were not identified in the CF_3_I-CO_2_ gas mixture breakdown tests. This means that these by-products are either not produced with a mixture of CF_3_I and CO_2_ or their production is reduced in quantity so that they are not detectable by the GCMS when CF_3_I is mixed with CO_2_ in a partial pressure mixture of 30% or less after 100 breakdown events. 

In the GCMS analysis of the CF_3_I-CO_2_ gas mixture after breakdown it is identified that additional by-products of C_2_F_6_O_3_ and potentially C_2_H_3_F_3_ and C_2_F_9_I are produced which are not detected in the analysis of pure CF_3_I after breakdown. These by-products are produced as a direct result of the interaction between CF_3_I and CO_2_ when used as a mixture which is subjected to arcing events of this energy level and are not produced when both CF_3_I or CO_2_ are used as an insulation/interruption medium which undergoes separate arcing events.

### 3.2. Discussion of 30:70% CF_3_I-CO_2_ GCMS Column Comparison

**Figure 5:**
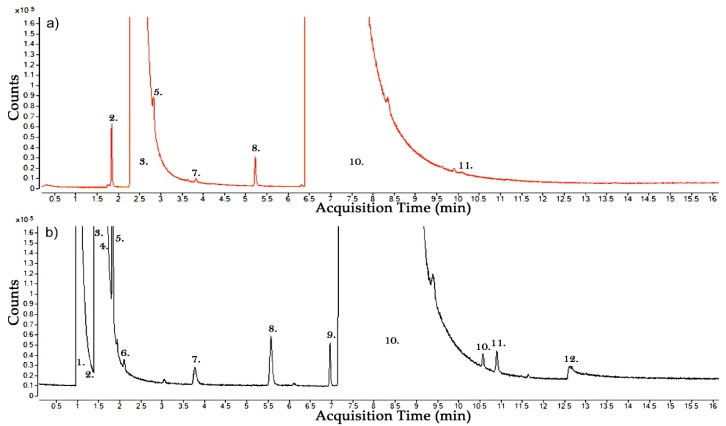
GCMS Analysis of the gaseous by-products of a 30:70% CF_3_I-CO_2_ gas mixture, (**a**) Orange line—gas analysis using a SilicaPlot column, (**b**) Black line—gas analysis using a GS GasPro column. 1. CO—Carbon Monoxide, 3. CO_2_—Carbon Dioxide, 5. CHF_3_—Fluoroform, 8. C_2_HF_5_—Ethane, pentafluoro-, 10. CF_3_I—Methane, trifluoroiodo-, 12. H_2_O—Water, 2. C_2_F_6_O_3_—Trioxide, bis(trifluoromethyl) or CF_4_—Tetrafluoromethane, 4. C_2_F_6_—Ethane, hexafluoro-, 6. C_2_F_6_O_3_—Trioxide, bis(trifluoromethyl), 7. C_3_F_8_—Perfluoropropane, 9. CF_4_—Tetrafluoromethane or C_2_F_6_O_3_—Trioxide, bis(trifluoromethyl) or C_2_H_3_F_3_—Ethane, 1,1,1-trifluoro-, 11. C_2_F_5_I—Pentafluoroethyliodide or C_2_F_9_I—Tetrafluoro(pentafluoroethyl)iodine.

A brief comparison for this paper was also conducted using two different GCMS columns. In Figure 5a the plot shows the gas chromatogram analysed by using a SiliPlot Column (Agilent, Amstelveen, Netherlands). In Figure 5b the plot shows the gas chromatogram analysed using a GS-GasPro Column (Agilent, Santa Clara, CA, USA). Although these two separate gas samples were analysed using different columns under relatively similar breakdown conditions to a 30%–70% CF_3_I-CO_2_ gas mixture some conclusions can be drawn about the use of these columns under this testing regime. 

It can be shown from Figure 5 that at the front end of the run light gases such as CO and CO_2_ are easier to distinguish using a GS-GasPro column as the separation of the column allows for individual identification which is not possible in the SilicaPlot column. It can also be shown that a gas with elution time similar to that of CF_3_I such as CF_4_ (No. 9. In Figure 5b) might also be harder to distinguish because these gases elute together and due to the high concentration of CF_3_I it is harder to distinguish CF_4_ at this retention time. It was therefore chosen to carry out all of the tests in the above sections using the GCMS with the GS-GasPro column.

## 4. Materials and Methods

**Figure 6:**
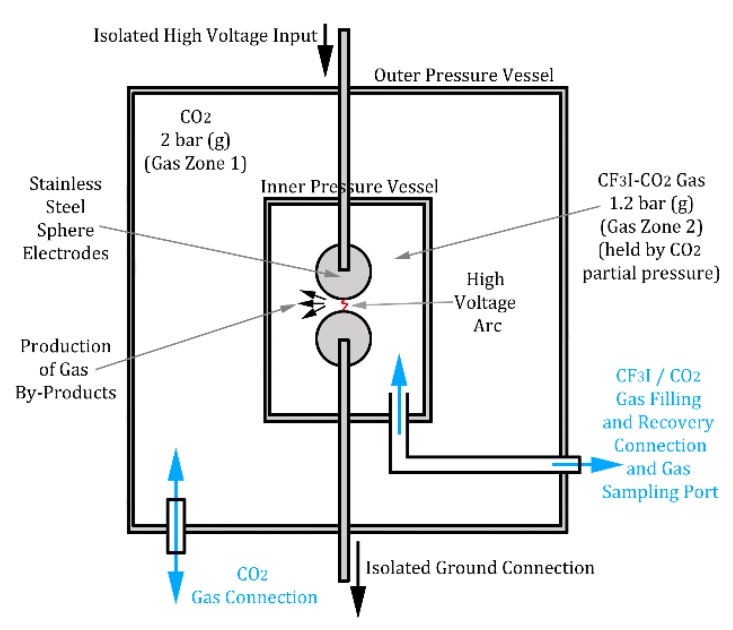
Gas Sample and Pressure Vessel Test Setup.

In this paper the test electrode setup was fixed as shown in Figure 6. In this setup the electrodes were of sphere-sphere geometry with a diameter of 25 mm and manufactured from stainless steel 304 which is composed of the primarily alloys: iron (Fe), Chromium (Cr) and Nickel (Ni) and various other trace elements [14]. Stainless steel 304 has a melting point of 1450 °C [14] so can only play a role in the decomposition products if the temperature in the arc reaches above this. These sphere electrodes are insulated using pure CO_2_, CF_3_I gas or a 30%:70% CF_3_I-CO_2_ gas mixture at a total pressure of 1.2 bar (g) which is contained within a pressurised tube and filled using stainless steel gas connectors. This insulated tube is connected to a high voltage bushing which is air insulated on one side and insulated by CO_2_ at 2 bar (g) on the inside. This CO_2_, which insulates the bushing, also surrounds the pressurised tube vessel containing the gas/gas mixture under test therefore keeping the test gas contained under a high pressure differential. This differential pressure eliminates any potential leakage as any leakage is more likely to occur from the CO_2_ vessel inwards rather than the CF_3_I gas tube outwards. The gases used have a purity level of >99.9% for CF_3_I [15] and 99.8% for CO_2_ [16]. The vessel containing CF_3_I is electrically isolated from the main vessel. The ground electrode for the CF_3_I vessel is connected via a dedicated grounding earth connection which is isolated from the outside pressure vessel walls, therefore ensuring the voltage and current measurements are separate from any external effects on the main vessel.

The gas mixture 30%–70% CF_3_I-CO_2_ was chosen to be analysed based on extensive research of the various characteristics of this gas mixture including electrical. In reference [9] the electrical characteristics of 10%–90%, 20%–80% and 30%–70% are discussed and it was found that 30%–70% had the best electrical performance. Another factor that greatly impacts the decision to use this gas mixture is the boiling point of this mixture which are limited by the restraints and specifications for indoor/outdoor high voltage electrical switchgear which states the operating temperatures of the equipment in the UK/Europe. 30%–70% CF_3_I-CO_2_ has a boiling point of −12.5 °C at a pressure of 0.5 MPa [17], while a higher percentage concentration of CF_3_I in the gas mixture would fall outside the guided operating temperatures for gas insulated switchgear specifications at this common operating pressure.

**Figure 7:**
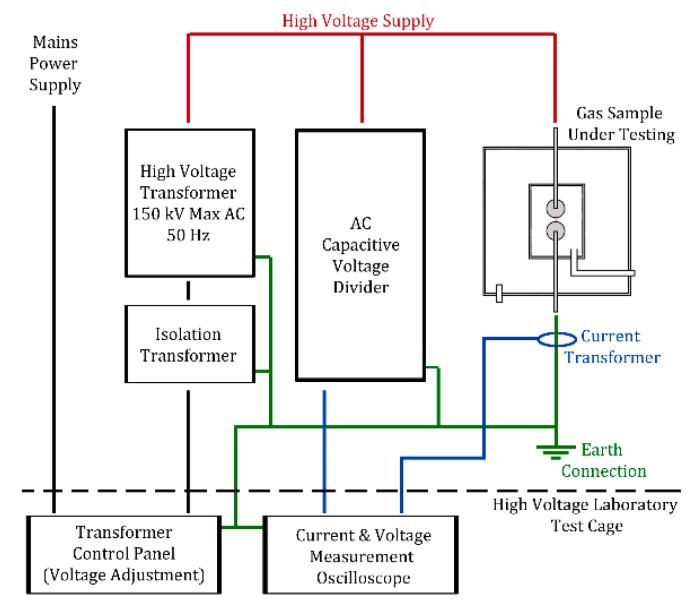
High Voltage Test Arrangement.

**Figure 8:**
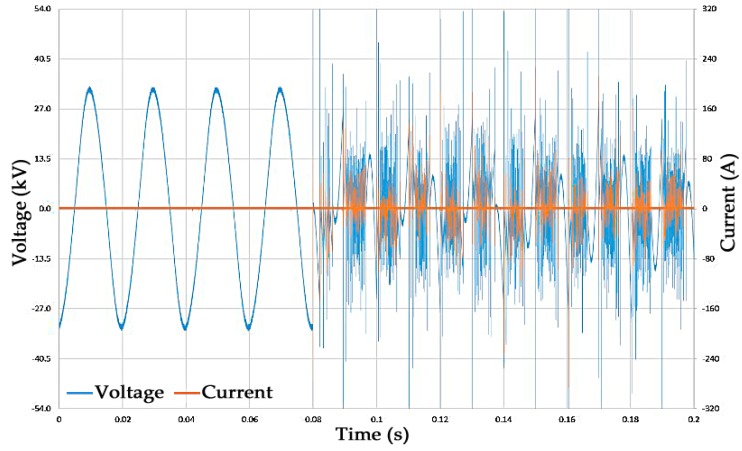
Voltage and current measurement of a breakdown event in CF_3_I at 1.2 bar (g).

The high voltage bushing of the gas pressure vessel is connected to a 150 kV rms AC transformer (Ferranti, Hollinwood, England) and a capacitive divider (Ferranti, Hollinwood, England) with a ratio of 6736:1 which allows for voltage measurements to be taken as shown in Figure 7. The current was measured upon breakdown of the gas gap using a current transformer (CT) (Stangenes Industries Inc, Palo Alto, CA, USA) which was connected around the grounding strap connected to the ground electrode. Each breakdown across the tested gas gap produces a separate voltage and current waveform which is recorded using an oscilloscope (Teledyne LeCroy, Glasgow, UK) triggered by the rise in current on the grounded electrode, an example of this is shown in Figure 8. For each event recorded the voltage on the output of the transformer is slowly increased until a breakdown occurs at which point it is lowered until the arc is self-extinguished by the gas. A constant measurement of the voltage applied to the test gas gap is also recorded using a secondary oscilloscope (Teledyne LeCroy), which helps determine a breakdown or no-breakdown scenario. When a breakdown occurs, the output voltage of the transformer drops because it has an earth fault allowing the flow of current to ground, under no-breakdown conditions the current is zero and the voltage is a steady oscillating 50 Hz AC waveform. 

After filling the pressurised gas vessel under test to 1.2 bar (g) a sample of the gas or gas mixture is taken using a stainless steel sampling tube. This is then connected to the GCMS which allows for different molecules to separate through the gas chromatography column and its mass spectrum to be analysed using the MS. The gas in the pressurised vessel is not replaced and therefore every time a sample is taken the gas pressure and density between the sphere gas gap is lowered, therefore, reducing the overall high voltage insulation strength. An initial sample of each gas/gas mixture is analysed using the GCMS to confirm its identity and identify whether any contamination exists before any high voltage arcing takes place, an example of this is shown in Figure 1, Figure 2 and Figure 3. After the initial sample is taken the gas sampling section is connected to the pressure vessel for the duration of the high voltage tests and only removed to allow the gas sample to be introduced to the GCMS. For each gas mixture the gas sampler and GCMS are flushed using a carrier gas of pure Helium which is 99.999% pure [18], therefore ensuring no-cross contamination takes place. During each run the GC oven is kept at a constant temperature of 50 °C for 5 min, the temperature is then ramped at 15 °C/min for 8 min and then kept at a constant temperature of 170 °C for a final 5 min leading to a total run time of 18 min. The GC is running the sample through for analysis in split mode with a ratio of 10:1. The MS is scanning for a mass between 5–350 with a step size of 0.1 *m*/*z* for the entire run.

For each test the vessel was flushed and vacuumed (<1 mbar [19]) and re-filled with CO_2_ five times before the final sample was introduced to remove cross contamination from the connecting hoses/vessel etc. For the pure CF_3_I test the vessel was flushed using helium and vacuumed fifteen times before the final sample of CF_3_I was introduced into the system. After each group of tests containing CF_3_I the gas/gas mixture was first recovered to a separate cylinder using a gas recovery system, thereby ensuring that the amount of CF_3_I released is minimal before the system was flushed. The experiments were conducted in the following order: pure CO_2_, 30%:70% CF_3_I-CO_2_ and then pure CF_3_I to ensure as few solid by-products were left inside the vessel which could affect the next set of results. The initial sample was taken following filling but preceding any high voltage breakdown. The sampling tube was then re-attached to the vessel and 50 breakdowns across the gas sample were conducted. The sample was then connected to the GCMS where the gas was analysed, this procedure was then repeated once 100 breakdown events had been undertaken when a final gas sample was taken before the gas/gas mixture was recovered into a separate cylinder. 

## 5. Conclusions

This paper experimentally evidences the use of CF_3_I and CF_3_I-CO_2_ gas mixtures in high voltage gas insulated equipment as an alternative insulation medium and examines the by-products produced when AC breakdown events occur through these gases. 

The experimental gas samples analysed using a GCMS show that in pure CF_3_I the by-products from arcing events are: CF_4_, C_2_F_6_, C_2_F_4_, C_3_F_8_, C_3_F_6_ or C_4_F_8_, C_2_F_5_I, C_3_F_7_I and C_3_F_7_IO with potential by-products of CHF_3_ and C_2_HF_5_. In a 30:70% CF_3_I-CO2 gas mixture the gas samples analysed showed by-products of: C_2_F_6_O_3_, C_2_F_6_, C_3_F_8_ and CF_4_ with potential by-products of C_2_H_3_F_3_, C_2_F_5_I or C_2_F_9_I, C_2_HF_5_ and CHF_3_.

It can be shown that prior to the breakdown tests being carried out, in both the pure CF_3_I and CF_3_I-CO_2_ gas samples, that both CHF_3_ and C_2_HF_3_ are detected so these are potential by-products, but it is difficult to conclusively prove this from these results. From both the pure CF_3_I and CF_3_I-CO_2_ breakdown tests it can be shown that CF_4_, C_2_F_6_, C_3_F_8_ and C_2_F_5_I are produced as gaseous by-products. In the pure CF_3_I breakdown tests, the gas analysis showed additional by-products of C_2_F_4_, C_3_F_6_, C_3_F_7_I, C_3_F_7_IO and potentially C_4_F_8_ which were not identified in the CF_3_I-CO_2_ gas mixture breakdown tests. In the GCMS analysis of the CF_3_I-CO_2_ gas mixture after breakdown it is identified that additional by-products of C_2_F_6_O_3_ and potentially C_2_H_3_F_3_ and C_2_F_9_I are produced which are not detected in the analysis of pure CF_3_I after breakdown, these by-products are produced as a direct result of the interaction between CF_3_I and CO_2_. All potential by-products of CF_3_I and CF_3_I-CO_2_ and their associated hazards and exposure controls are detailed more extensively in Table A1.

It is necessary to point out that if CF_3_I reacted with any of the materials used in the filling valve, hose, pressure vessel wall or gas connections/sampling port during its 10 hour filling time that these by-products may be included in these results. It is also important to note that iodine is produced in a semi-solid state from arcing events in CF_3_I gas mixtures as shown in reference [20].

Future work will involve further analysis into the gaseous by-products of CF_3_I gas mixture with other potential buffer gases.

## Figures and Tables

**Table 1:** GCMS analysis of the gaseous by-products of pure CF_3_I and a 30%:70% CF_3_I-CO_2_ gas mixture.

By-product Labelled Number in Figure 4	CF_3_I and CF_3_I-CO_2_ Gas Sample–Detected before Breakdown	CF_3_I and CF_3_I-CO_2_ Gas Sample–By-products Detected after Breakdown	CF_3_I Gas Sample only–By-products Detected after Breakdown	CF_3_I-CO_2_ Gas Sample only–By-products Detected after Breakdown
**1**	CO—Carbon Monoxide			
**2**		CF_4_—Tetrafluoromethane		CF_4_ or C_2_F_6_O_3_—Trioxide, bis(trifluoromethyl)
**3**	CO_2_—Carbon Dioxide			
**4**		C_2_F_6_—Ethane, hexafluoro-		
**5**			C_2_F_4_—Ethene, tetrafluoro-	
**6**	CHF_3_—Fluoroform			
**7**				C_2_F_6_O_3_—Trioxide, bis(trifluoromethyl)
**8**		C_3_F_8_—Perfluoropropane		
**9**	C_2_HF_5_—Ethane, pentafluoro			
**10**			C_3_F_6_ or C_4_F_8_—Cyclobutane, octafluoro-	
**11**				C_2_F_6_O_3_ or C_2_H_3_F_3_—Ethane, 1,1,1-trifluoro-
**12**	CF_3_I—Methane, trifluoroiodo-			
**13**		C_2_F_5_I—Pentfluoroethyliodide		C_2_F_5_I or C_2_F_9_I—Tetrafluoro(pentafluoroethyl)iodine
**14**	H_2_O—Water			
**15**	I_2_—Iodine (CF_3_I test only)			
**16**			C_3_F_7_I—Perfluoropropyl iodide	
**17**			C_3_F_7_IO—Tetrafluoro-1 trifluoromethoxy 1-iodoethane	

## Appendix A

**Table A1:** Potential By-Products of CF_3_I and CF_3_I-CO_2_ gas mixtures and their associated hazards and exposure controls. Note: data retrieved was correct at time of writing 19/03/2019, however, it is subject to frequent change and re-evaluation.

Gas Detected	CAS Number	Molecular Weight	Labels	Exposure Limits	Exposure Controls	Reference
CO_2_—Carbon Dioxide	CAS-No. 124-38-9 EC No. 204-696-9	44.01 g/mol	H280: Contains gas under pressure; may explode if heated.	TWA 5000 ppm 9150 mg/m^3^ STEL 15,000 ppm 27,400 mg/m^3^ TWA 5000 ppm 9000 mg/m^3^	P403: Store in a well-ventilated place.	[21]
CF_3_I—Methane, trifluoroiodo-	CAS-No.: 2314-97-8 EC-No.: 219-014-5 Index-No.: 602-086-00-0	195.91 g/mol	H341 Suspected of causing genetic defects.	LCLo inhalation 1 pph/4H (10,000 mg/kg)	P280 Wear protective gloves/ protective clothing/ eye protection/ face protection. P410 Protect from sunlight. P502 Refer to manufacturer/ supplier for information on recovery/ recycling	[15] [22,23]
CO—Carbon Monoxide	CAS-No. 630-08-0 EC-No. 211-128-3 Index-No. 006-001-00-2	28.01 g/mol	H220 Extremely flammable gas. H331 Toxic if inhaled. H360D May damage the unborn child. H372 Causes damage to organs through prolonged or repeated exposure.	STEL 200 ppm TWA 30 ppm LC50 Inhalation-Rat-4 h-1807 ppm	Avoid contact with skin, eyes and clothing. Wash hands before breaks and immediately after handling the product.	[24]
CF_4_—Tetrafluoromethane	CAS number: 75-73-0	88.01 g/mol	H280: Contains gas under pressure; may explode if heated. H315: Causes skin irritation. H319: Causes serious eye irritation. H335: May cause respiratory irritation.	No occupational exposure limit. DNEL not available PNEC not available.	P271: Use only outdoors or in a well-ventilated area. P280: Wear protective gloves/protective clothing/eye protection/face protection. R36/37/38: Irritating to eyes, respiratory system and skin. R44: Risk of explosion if heated under confinement. S36/37/39: Wear suitable protective clothing, gloves and eye/face protection. S45: In case of accident or if you feel unwell, seek medical advice immediately	[25,26]
C_2_F_6_—Ethane, hexafluoro-	CAS number: 76-16-4 EINECS number: 200-939-8	138.01 g/mol	H315: Causes skin irritation. H319: Causes serious eye irritation. H335: May cause respiratory irritation.	LC inhalation 20 pph/2H (200,000 mg/kg) LD50 4400 mg/kg	P261: Avoid breathing gas. P271: Use only outdoors or in a well-ventilated area. P280: Wear protective gloves/protective clothing/eye protection/face protection.	[27]
C_2_F_4_—Ethene, tetrafluoro-	CAS No.: 116-14-3 EC No. 204-126-9 UN No. 1081	100.02 g/mol	H220 (90.55%): Extremely flammable gas [Danger Flammable gases] H350 (85.07%): May cause cancer [Danger Carcinogenicity] H371 (73.63%): May cause damage to organs [Warning Specific target organ toxicity, single exposure]	LC50 inhalation 40,000 ppm/4H (40,000 mg/kg) LC50 inhalation143 gm/m^3^/4H (143,000 mg/kg) LC50inhalation 116 gm/m^3^/4H (116,000 mg/kg)	P201 Obtain special instructions before use, P210 Keep away from heat, hot surface, sparks, open flames and other ignition sources. - No smoking, P260 Do not breathe dust/fume/gas/mist/vapors/spray, P281 Use personal protective equipment as required, P308 + P313, P309 + P311 if exposed or if you feel unwell Call a POISON CENTER or doctor, P377, P381, P403 Store in a well-ventilated place, P405, P410 + P403 Protect from sunlight.	[28]
CHF_3_—Fluoroform	CAS number: 75-46-7 EINECS number: 200-872-4	70.01 g/mol	H315: Causes skin irritation. H319: Causes serious eye irritation. H335: May cause respiratory irritation.	LC50 > 200,000 ppm/2H LC50 > 663,000 ppm/4H	P271: Use only outdoors or in a well-ventilated area. P280: Wear protective gloves/protective clothing/eye protection/face protection. R36/37/38: Irritating to eyes, respiratory system and skin. S36/37/39: Wear suitable protective clothing, gloves and eye/face protection. S45: In case of accident or if you feel unwell, seek medical advice immediately (show the label where possible	[29,30]
C_2_F_6_O_3_—Trioxide, bis(trifluoromethyl)	CAS No. 1718-18-9	186.01 g/mol	No data available.	No data available.	No data available.	[31]
C_3_F_8_—Perfluoropropane	CAS number: 76-19-7 EINECS number: 200-941-9	188.02 g/mol	H280: Contains gas under pressure; may explode if heated. H319: Causes serious eye irritation. H335: May cause respiratory irritation.	LD 20 mL/kg (20 mg/kg)	P260: Do not breathe gas. P271: Use only outdoors or in a well-ventilated area. P280: Wear protective gloves/protective clothing/eye protection/face protection.	[32]
C_2_HF_5_—Ethane, pentafluoro	CAS number: 354-33-6 EC No. 206-557-8	120.02 g/mol	H280: Contains gas under pressure; may explode if heated. H315: Causes skin irritation. H319: Causes serious eye irritation. H335: May cause respiratory irritation.	LC50 inhalation 2910 gm/m^3^/4H (2910,000 mg/kg) LC50inhalation 2735 gm/m^3^/2H (2,735,000 mg/kg)	P260: Do not breathe gas. P271: Use only outdoors or in a well-ventilated area. P280: Wear protective gloves/protective clothing/eye protection/face protection.	[33,34]
C_3_F_6_—Hexafluoropropene	CAS-No.: 116-15-4 EC-No.: 204-127-4 Index-No.: 602-061-00-4	150.02 g/mol	H332 Harmful if inhaled. H335 May cause respiratory irritation. H371 May cause damage to organs. H373 May cause damage to organs through prolonged or repeated exposure.	LC50 750 ppm/4H LC16 5200 mg/m^3^/2H LC50 1600 mg/m^3^/2H LC84 13,400 mg/m^3^/2H	P260 Do not breathe dust/fume/gas/mist/vapours/spray. P308 + P311 IF exposed or concerned: Call a POISON CENTER/doctor. P410 + P403 Protect from sunlight. Store in a well-ventilated place.	[35,36]
C4F_8_—Cyclobutane, octafluoro-	CAS number: 115-25-3 EINECS number: 204-075-2	200.03 g/mol	H315: Causes skin irritation. H319: Causes serious eye irritation. H335: May cause respiratory irritation. H280: Contains gas under pressure; may explode if heated.	LCLo inhalation 78 pph/2H (780,000 mg/kg)	P271: Use only outdoors or in a well-ventilated area. P261: Avoid breathing gas. P280: Wear protective gloves/protective clothing/eye protection/face protection.	[37]
C_2_H_3_F_3_—Ethane, 1,1,1-trifluoro-	CAS number: 420-46-2	84.04 g/mol	H220: Extremely flammable gas. H315: Causes skin irritation. H319: Causes serious eye irritation. H335: May cause respiratory irritation.	LC50 inhalation 54 pph/4H (540,000 mg/kg)	P210: Keep away from heat, hot surfaces, sparks, open flames and other ignition sources. No smoking. P280: Wear protective gloves/protective clothing/eye protection/face protection.	[38,39]
C_2_F_5_I—Pentfluoroethyliodide	CAS-No.: 354-64-3 EC-No.: 206-566-7	245.92 g/mol	H315 Causes skin irritation. H319 Causes serious eye irritation. H335 May cause respiratory irritation.	LC inhalation 40,000 ppm/4H (40,000 mg/kg)	P261 Avoid breathing dust/fume/gas/mist/vapours/spray. P305 + P351 + P338 IF IN EYES: Rinse cautiously with water for several minutes. Remove contact lenses, if present and easy to do. Continue rinsing. P410 + P403 Protect from sunlight. Store in a well-ventilated place. EUH044 Risk of explosion if heated under confinement. 2	[40,41]
C_2_F_9_I—Tetrafluoro(pentafluoroethyl)iodine	CAS-No.: 20636-76-4	321.91 g/mol	No data available	No data available	No data available	[42]
I_2_—Iodine (CF_3_I test only)	CAS-No.: 7553-56-2 EC-No.: 231-442-4 Index-No.: 053-001-00-3	253.81 g/mol	H312 + H332 Harmful in contact with skin or if inhaled. H315 Causes skin irritation. H319 Causes serious eye irritation. H335 May cause respiratory irritation. H372 Causes damage to organs (Thyroid) through prolonged or repeated exposure if swallowed. H400 Very toxic to aquatic life.	STEL 0.1 ppm LD50 Oral-Rat-14,000 mg/kg LC50 Inhalation-Rat-4 h- > 4.588 mg/L LC50 Dermal-Rat-male-1425 mg/kg	P261 Avoid breathing dust. P273 Avoid release to the environment. P280 Wear protective gloves/protective clothing. P305 + P351 + P338 IF IN EYES: Rinse cautiously with water for several minutes. Remove contact lenses, if present and easy to do. Continue rinsing. P314 Get medical advice/attention if you feel unwell.	[43]
C_3_F_7_I—Perfluoropropyl iodide	CAS-No.: 754-34-7 EC-No.: 212-045-5	295.93 g/mol	H315 (100%): Causes skin irritation H319 (100%): Causes serious eye irritation H335 (75%): May cause respiratory irritation [Warning Specific target organ toxicity, single exposure; Respiratory tract irritation]	LC50 Inhalation-2 h-404,000 mg/m^3^	P261 Avoid breathing dust/fume/gas/mist/vapors/spray, P271 Use only outdoors or in a well-ventilated area, P280 Wear protective protection, P302 + P352 If on skin wash with plenty of water, P304 + P340 If inhaled remove victim to fresh air, P305 + P351 If in eyes rinse cautiously with water for several minutes, P312 Call a poison centre or doctor if you feel unwell, P321, P362 Take off contaminated clothing, P403 + P233 Store in a well-ventilated place, P405 Store locked up.	[44,45]
C_3_F_7_IO—Tetrafluoro-1 trifluoromethoxy 1-iodoethane 1,2,2,2-Tetrafluoro-1-iodoethyl trifluoromethyl ether	CAS-No.: 139604-89-0	311.92 g/mol	H302 Harmful if swallowed [Warning Acute toxicity, oral] H312 Harmful in contact with skin. H315 Causes skin irritation H319 Causes serious eye irritation H332 Harmful if inhaled H335 May cause respiratory irritation [Warning Specific target organ toxicity, single exposure; Respiratory tract irritation]	No data available	P261 Avoid breathing dust/fume/gas/mist/vapors/spray, P264 Wash thoroughly after handling, P271 Use only outdoors or in a well-ventilated area, P280 Wear protective protection, P301 + P312 f swallowed call a poison centre if you feel unwell, P302 + P352 If on skin wash with plenty of water, P304 + P312 If inhaled call a poison centre or doctor if you feel unwell, P304 + P340 If inhaled remove victim to fresh air, P305 + P351 + P338 If in eyes rinse cautiously with water for several minutes, P312 Call a POISON CENTER or doctor if you feel unwell, P321, P322, P330 Rinse mouth, P332 + P313 If skin irritation occurs get medical advice/attention, P337 + P313 If eye irritation persists get medical advice/attention, P362 Take off contaminated clothing, P363 Wash contaminated clothing before reuse, P403 + P233 Store in a well-ventilated place & Keep container tightly closed, P405 Store locked up.	[46]

## References

[1] Widger P., Haddad A.M. (2018). Evaluation of SF_6_ Leakage from Gas Insulated Equipment on Electricity Networks in Great Britain. Energies.

[2] Myhre G., Shindell D., Bréon F.M., Collins W., Fuglestvedt J., Jianping H., Koch D., Lamarque J.F., David L., Mendoza B. (2013). Anthropogenic and Natural Radiative Forcing.

[3] Intergovernmental Panel on Climate Change (IPCC) (2007). Working Group I Contribution to Fourth Assessment Report of the Intergovernmental Panel on Climate Change.

[4] National Grid (2016/2017). National Grid Electricity Transmission Network Innovation Allowance Annual Summary. https://www.nationalgrid.com/sites/default/files/documents/National%20Grid%20Electricity%20Transmission%20NIA%20Annual%20Summary%202016-17.pdf.

[5] ABB AirPlus TM: An Alternative to SF_6_ as an Insulation and Switching Medium in Electrical Switchgear. https://library.e.abb.com/public/3405a31190934a8c98997eca8fc811be/ABB%20Review%202-2016_AirPlus_An%20Alternative%20to%20SF6.pdf.

[6] Beroual A., Haddad A. (2017). Recent Advances in the Quest for a New Insulation Gas with a Low Impact on the Environment to Replace Sulphur Hexafluoride (SF_6_) Gas in High-Voltage Power Network Applications. Energies.

[7] Widger P., Haddad A., Griffiths H. (2016). Breakdown performance of vacuum circuit breakers using alternative CF_3_I-CO_2_ insulation gas mixture. IEEE Trans. Dielectr. Electr. Insul..

[8] Chen L., Widger P., Kamarudin M.S., Griffiths H., Haddad A. (2017). CF_3_I Gas Mixtures: Breakdown Characteristics and Potential for Electrical Insulation. IEEE Trans. Power Deliv..

[9] Widger P., Griffiths H., Haddad A. (2018). Insulation strength of CF_3_I-CO_2_ gas mixtures as an alternative to SF_6_ in MV switch disconnectors. IEEE Trans. Dielectr. Electr. Insul..

[10] Li Y., Zhang X., Zhang J., Xiao S., Xie B., Chen D., Gao Y., Tang J. (2019). Assessment on the toxicity and application risk of C_4_F_7_N: A new SF_6_ alternative gas. J. Hazard. Mater..

[11] Rabie M., Franck C.M. (2018). Assessment of Eco-friendly Gases for Electrical Insulation to Replace the Most Potent Industrial Greenhouse Gas SF_6_. Environ. Sci. Technol..

[12] Zhang X., Li Y., Xiao S., Tang J., Tian S., Deng Z. (2017). Decomposition Mechanism of C_5_F_10_O: An Environmentally Friendly Insulation Medium. Environ. Sci. Technol..

[13] Kamarol M., Nakayama Y., Hara T., Ohtsuka S., Hikita M. Gas Decomposition Analysis of CF_3_I under AC Partial Discharge of Non-uniform Electric Field Configuration. Proceedings of the 10th Japan-Korea Joint Symposium on Electrical Discharge and High Voltage Engineering, Shibaura Institute of Technology.

[14] Aalco (2018). Stainless Steel: 1.4301 Sheet and Plate. http://www.aalco.co.uk/online-tools/product-finder/product.aspx?productid=2083.

[15] Tosoh Corporation, (Tosoh Corporation, Tokyo, Japan) (2011). CF_3_I (Trifluoroiodomethane) Safety data sheet according to Regulation (EC) No. 1907/2006, Cas No. 2314-97-8. Personal communication.

[16] (2017). BOC, Carbon dioxide Factsheet. https://www.boconline.co.uk/en/legacy/attachment?files=tcm:t410-54560,tcm:410-54560,tcm:10-54560.

[17] Kasuya H., Katagiri H., Kawamura Y., Saruhashi D., Nakamura Y., Mizoguchi H., Yanabu S. Measurement of Decomposed Gas Density of CF_3_I-CO2 Mixture. Proceedings of the 16th International Symposium on High Voltage Engineering (ISH 2009), South African Inst. of Electrical Engineers.

[18] (2018). BOC, Helium Purity level. https://www.boconline.co.uk/shop/en/uk/helium--cp-grade--300bar-cylinder-171698#product1.

[19] Vacuum pump final vacuum level, Dilo B078R06 portable vacuum pump unit specifications, Dilo GmbH, Babenhausen, 2012 Product Catalogue. https://www.dilo-gmbh.com/fileadmin/user_upload/8._Downloads/2._englisch/2._SF6-Gashandling/2._SF6-Servicegeraete/Mini_Series_-_modular.pdf.

[20] Widger P., Haddad A. (2017). Solid by-products of a CF_3_I-CO_2_ insulating gas mixtures on electrodes after lightning impulse breakdown. J. Phys. Commun..

[21] (2017). BOC, Carbon Dioxide Materials Safety Data Sheet. https://www.boconline.co.uk/en/legacy/attachment?files=tcm:4410-39607,tcm:410-39607,tcm:10-39607.

[22] Sigma Aldrich, CF_3_I Material Safety Data Sheet. https://www.sigmaaldrich.com/MSDS/MSDS/DisplayMSDSPage.do?country=GB&language=en&productNumber=171441&brand=ALDRICH&PageToGoToURL=https%3A%2F%2Fwww.sigmaaldrich.com%2Fcatalog%2Fproduct%2FALDRICH%2F171441%3Flang%3Den.

[23] U.S. National Library of Medicine National Center for Biotechnology Information, Pubchem, CF_3_I. https://pubchem.ncbi.nlm.nih.gov/compound/16843#section=Top.

[24] Sigma Aldrich, CO Material Safety Data Sheet. https://www.sigmaaldrich.com/MSDS/MSDS/DisplayMSDSPage.do?country=GB&language=en&productNumber=295116&brand=ALDRICH&PageToGoToURL=https%3A%2F%2Fwww.sigmaaldrich.com%2Fcatalog%2Fproduct%2Faldrich%2F295116%3Flang%3Den.

[25] Apollo Scientific, CF_4_ Material Safety Data Sheet. http://www.apolloscientific.co.uk/downloads/msds/PC1650_msds.pdf.

[26] BOC Online, CF_4_ Material Safety Data Sheet. https://www.boconline.co.uk/en/images/sg_116_tetrafluoromethane_r14_tcm410-64642.pdf.

[27] Apollo Scientific, C_2_F_6_ Material Safety Data Sheet. http://www.apolloscientific.co.uk/downloads/msds/PC7201_msds.pdf.

[28] U.S. National Library of Medicine National Center for Biotechnology Information, Pubchem, C_2_F_4_. https://pubchem.ncbi.nlm.nih.gov/compound/8301#section=Top.

[29] Apollo Scientific, CHF_3_ Material Safety Data Sheet. http://www.apolloscientific.co.uk/downloads/msds/PC7400_msds.pdf.

[30] U.S. National Library of Medicine National Center for Biotechnology Information, Pubchem, CHF3. https://pubchem.ncbi.nlm.nih.gov/compound/6373#section=Top.

[31] U.S. National Library of Medicine National Center for Biotechnology Information, Pubchem, C_2_F_6_O_3_. https://pubchem.ncbi.nlm.nih.gov/compound/549862#section=Top.

[32] Apollo Scientific, C_3_F_8_ Material Safety Data Sheet. http://www.apolloscientific.co.uk/downloads/msds/PC6210_msds.pdf.

[33] Apollo Scientific, C_2_HF_5_ Material Safety Data Sheet. http://www.apolloscientific.co.uk/downloads/msds/PC5590_msds.pdf.

[34] U.S. National Library of Medicine National Center for Biotechnology Information, Pubchem, C_2_HF_5_. https://pubchem.ncbi.nlm.nih.gov/compound/9633#section=Top.

[35] Sigma Aldrich, C_3_F_6_ Material Safety Data Sheet. https://www.sigmaaldrich.com/MSDS/MSDS/DisplayMSDSPage.do?country=GB&language=en&productNumber=295388&brand=ALDRICH&PageToGoToURL=https%3A%2F%2Fwww.sigmaaldrich.com%2Fcatalog%2Fproduct%2Faldrich%2F295388%3Flang%3Den.

[36] U.S. National Library of Medicine National Center for Biotechnology Information, Pubchem, C_3_F_6_. https://pubchem.ncbi.nlm.nih.gov/compound/8302#section=Top.

[37] U.S. National Library of Medicine National Center for Biotechnology Information, Pubchem, C_4_F_8_. https://pubchem.ncbi.nlm.nih.gov/compound/8263#section=Top.

[38] Apollo Scientific, C_2_H_3_F_3_ Material Safety Data Sheet. http://www.apolloscientific.co.uk/downloads/msds/PC7298_msds.pdf#.

[39] U.S. National Library of Medicine National Center for Biotechnology Information, Pubchem, C_2_H_3_F_3_. https://pubchem.ncbi.nlm.nih.gov/compound/9868#section=Top.

[40] Sigma Aldrich, C_2_F_5_I Material Safety Data Sheet. https://www.sigmaaldrich.com/MSDS/MSDS/DisplayMSDSPage.do?country=GB&language=en&productNumber=331015&brand=ALDRICH&PageToGoToURL=https%3A%2F%2Fwww.sigmaaldrich.com%2Fcatalog%2Fproduct%2FALDRICH%2F331015%3Flang%3Den.

[41] U.S. National Library of Medicine National Center for Biotechnology Information, Pubchem, C_2_F_5_I. https://pubchem.ncbi.nlm.nih.gov/compound/9636#section=Top.

[42] U.S. National Library of Medicine National Center for Biotechnology Information, Pubchem, C_2_F_9_I. https://pubchem.ncbi.nlm.nih.gov/compound/550350#section=Top.

[43] Sigma Aldrich, I_2_ Material Safety Data Sheet. https://www.sigmaaldrich.com/MSDS/MSDS/DisplayMSDSPage.do?country=GB&language=en&productNumber=229695&brand=ALDRICH&PageToGoToURL=https%3A%2F%2Fwww.sigmaaldrich.com%2Fcatalog%2Fproduct%2Faldrich%2F229695%3Flang%3Den.

[44] Sigma Aldrich, C_3_F_7_I Material Safety Data Sheet. https://www.sigmaaldrich.com/MSDS/MSDS/DisplayMSDSPage.do?country=GB&language=en&productNumber=P10402&brand=ALDRICH&PageToGoToURL=https%3A%2F%2Fwww.sigmaaldrich.com%2Fcatalog%2Fproduct%2Faldrich%2Fp10402%3Flang%3Den.

[45] U.S. National Library of Medicine National Center for Biotechnology Information, Pubchem, C_3_F_7_I. https://pubchem.ncbi.nlm.nih.gov/compound/33977#section=Top.

[46] U.S. National Library of Medicine National Center for Biotechnology Information, Pubchem, C_3_F_7_IO. https://pubchem.ncbi.nlm.nih.gov/compound/550245#section=Top.

